# Validity and Reliability of the Swedish Version of the Gugging Swallowing Screen for use in Acute Stroke Care

**DOI:** 10.1007/s00455-024-10717-y

**Published:** 2024-05-16

**Authors:** Jenny Selg, Thorbjörn Holmlund, Eva Levring Jäghagen, Jenny McGreevy, Sara Svanberg, Per Wester, Patricia Hägglund

**Affiliations:** 1 Speech and Language Pathology, Department of Clinical Sciences, Faculty of Medicine, Umeå University, Umeå, Sweden; 2 Otorhinolaryngology, Department of Clinical Sciences, Faculty of Medicine, Umeå University, Umeå, Sweden; 3 Oral and Maxillofacial Radiology, Department of Odontology, Faculty of Medicine, Umeå University, Umeå, Sweden; 4 Department of Dietetics, Region Sörmland, Nyköping, Sweden; 5 Centre for Clinical Research Sörmland, Uppsala University, Eskilstuna, Sweden; 6 Stroke Unit, Umeå University Hospital, Umeå, Sweden; 7 Department of Clinical Sciences, Danderyd Hospital, Karolinska Institutet, Stockholm, Sweden; 8 Department of Public Health and Clinical Medicine, Umeå University, Umeå, Sweden

**Keywords:** Stroke, Dysphagia screening, Validation, Sensitivity, Specificity, The Gugging swallowing screen

## Abstract

The purpose of this study was to cross-culturally validate the Swedish version of the Gugging Swallowing Screen (GUSS-S) for use in the acute phase of stroke. Further, to evaluate the inter-rater reliability between different healthcare professionals. GUSS was translated into Swedish using a forward-backward method followed by expert rating to obtain content validity. For criterion validity, the GUSS-S score was compared with Flexible Endoscopic Evaluation of Swallowing (FEES) assessed with the Penetration-Aspiration Scale (PAS) in acute stroke patients (≤ 96 h after stroke onset). Convergent validity was calculated by comparison with the Functional Oral Intake Scale (FOIS) as per the comprehensive FEES assessment, the Standardized Swallowing Assessment (SSA), and the National Institutes of Health Stroke Scale (NIHSS). To evaluate inter-rater reliability, a nurse and a speech-language pathologist (SLP) independently assessed 30 patients. In total, 80 patients (32 women, median age 77 years (range 29–93) were included, mean 1.7 ± 0.9 days after admission. With a cut-off value of 14 points, the GUSS-S identified aspiration with a sensitivity of 100% and a specificity of 73% (area under the curve: 0.87, 95% CI 0.78–0.95). Spearman rank correlation showed very strong correlation between the GUSS-S and PAS (r_s_=-0.718, *P* = < 0.001) and FOIS (r_s_=0.720, *P* = 0.001) and strong correlation between the GUSS-S and SSA (r_s_=0.545, *P* = < 0.001) and NIHSS (r_s_=-0.447, *P* = 0.001). The inter-rater agreement for GUSS-S was substantial (_Kw_=0.67, *P = <* 0.001). The results indicate that the GUSS-S is a valid and reliable tool for the assessment of dysphagia in acute stroke patients by different healthcare professionals.

**Supplementary Information:**

The online version contains supplementary material available at 10.1007/s00455-024-10717-y.

## Introduction

Dysphagia, or swallowing difficulties, is a highly prevalent complication after stroke, with an estimated rate of up to 78% in the acute phase of stroke, with incidence varying with time and method of assessment [1]. The consequences of post-stroke dysphagia are partly related to restrictions of oral intake, resulting in malnutrition, dehydration, social isolation, and decreased quality of life. Moreover, dysphagia is related to a risk of aspiration, which can lead to choking or coughing while eating and/or drinking, and aspiration pneumonia [2]. To prevent these complications, international and national guidelines for stroke care recommend that all stroke patients should be formally screened for dysphagia [3, 4]. Should the patient show signs of aspiration during screening, the patient is usually kept nil-by-mouth until a more thorough assessment can be conducted. Access to specialized care, such as speech and language pathology, is often limited in more rural areas [5]. This may lead to the patient being nil-by-mouth for long periods after screening before further assessment can be conducted. If patients at risk of dysphagia can be identified at an early stage, aspiration pneumonia and other severe consequences can be substantially avoided [6].

Several types of assessment may be used to identify the presence of dysphagia. These include screening, clinical assessment, and instrumental assessment of swallowing, with all methods having a different purpose and reliability [7]. Instrumental assessments are considered the gold standard for detecting dysphagia and include, most commonly, Flexible Endoscopic Evaluation of Swallowing (FEES) and Videofluoroscopic Study of Swallowing (VFSS) [8]. These methods are performed by specialists to examine the patient’s swallowing function by visualizing the swallowing directly to confirm or reject a dysphagia diagnosis and/or plan treatment. Less in-depth than instrumental assessment, but still performed by a specialist, are clinical examinations of swallow focusing on assessing the safety and efficacy of the patient’s swallowing ability without direct visualization. Lastly, screening is used in the majority of patients to identify risk for dysphagia at an early stage. Screening should be quick, cost-effective, and with low risk, as well as user-friendly, since it is predominantly performed, in Sweden, by nursing staff without specialized knowledge of dysphagia [4]. A good screening method should be sensitive enough to detect all patients at risk of dysphagia/aspiration but at the same time specific enough to avoid false positives and unnecessary restrictions of intake or long periods of nil-by-mouth, prior to assessment by a speech-language pathologist (SLP). A sensitivity of 70% and a specificity of 60% have been suggested as acceptable values for dysphagia screening [9]. For nursing staff, a user-friendly and accurate screening method is an important tool to facilitate adherence to stroke care guidelines and can significantly improve the care given to the individual patient [4].

In Swedish hospitals, dysphagia screening is usually carried out using a water-swallowing test [10]. The most used screening tool on Swedish stroke units is the Standardized Swallowing Assessment, which is available in Swedish (SSA-S [11–13]), however, it has not been validated against the gold standard assessments VFSS or FEES. Additionally, water-swallowing tests may pose a risk of both over- and underdiagnosing the patient, since the screening often leads to refraining from oral feeding completely when only one consistency has been assessed. In the stroke population, patients may aspirate thin liquids but manage other viscosities well [14]. Conversely, other patients may experience difficulties with thicker consistencies but not with water, thus rendering the water-only swallowing test less suitable for determining appropriate oral intake.

To obtain a more nuanced view of the patient’s swallowing ability at screening, the Gugging Swallowing Screen (GUSS) can be used [15]. The GUSS involves a step-wise approach using liquid, semi-solid, and solid consistencies. The GUSS also considers other clinical indicators of risk for aspiration, such as vigilance, drooling, and ability to voluntarily cough, making it a more thorough screening tool. Depending on scoring, the GUSS screening tool also provides suggestions for dietary restrictions and recommendations for SLP referral, if necessary.

The GUSS is not currently available in Swedish and no screening methods currently used in Sweden have been validated against FEES or VFSS. The inter-rater reliability for the GUSS has also not been previously evaluated by comparing its use by different healthcare professionals, e.g., SLPs and nurses.

The purpose of this study was to conduct a cross-cultural validation of the Swedish version of the Gugging Swallowing Screen (GUSS-S) for use in the acute phase of stroke. An additional purpose was to evaluate the inter-rater reliability of the screening tool between different healthcare professionals. The hypothesis was that the GUSS-S would have high sensitivity and specificity, as well as high inter-rater reliability, which would be in line with previous validations of the tool [15–18].

## Method

### Study Design

**Fig. 1:**
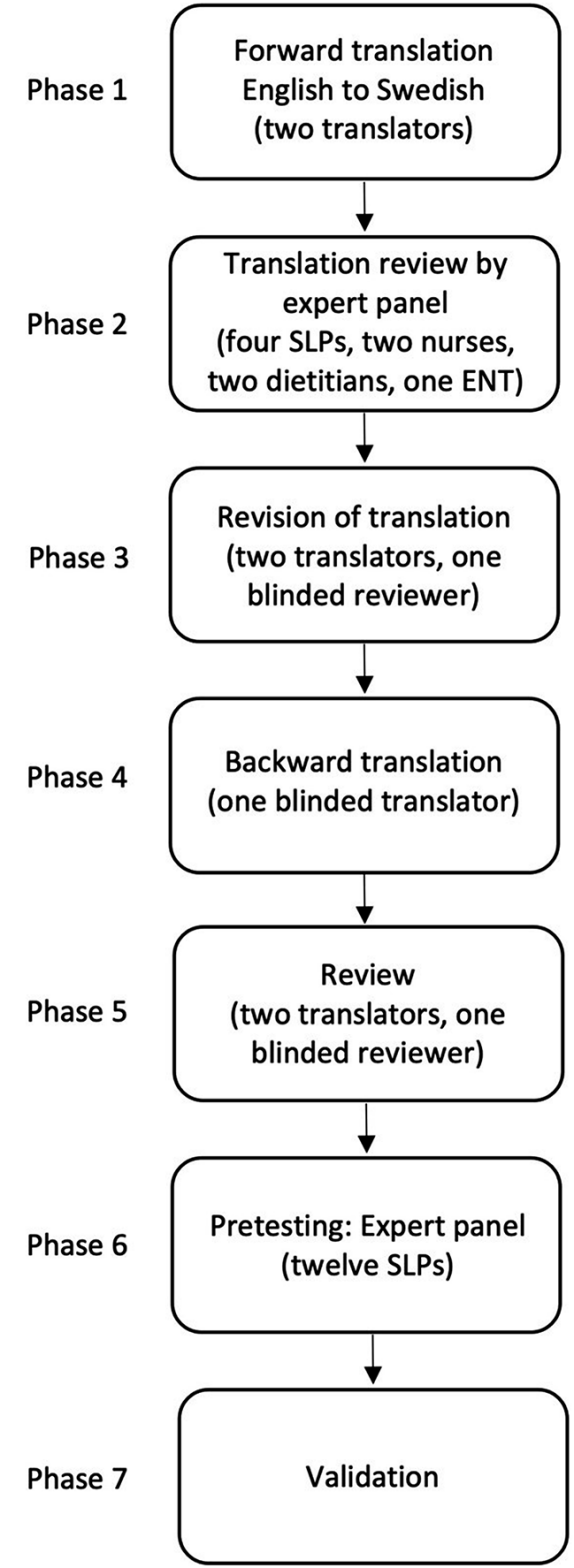
Flow chart of translation procedure. *Abbreviations* SLP = Speech Language Pathologist, ENT = Ear, Nose and Throat Doctor

**Fig. 2:**
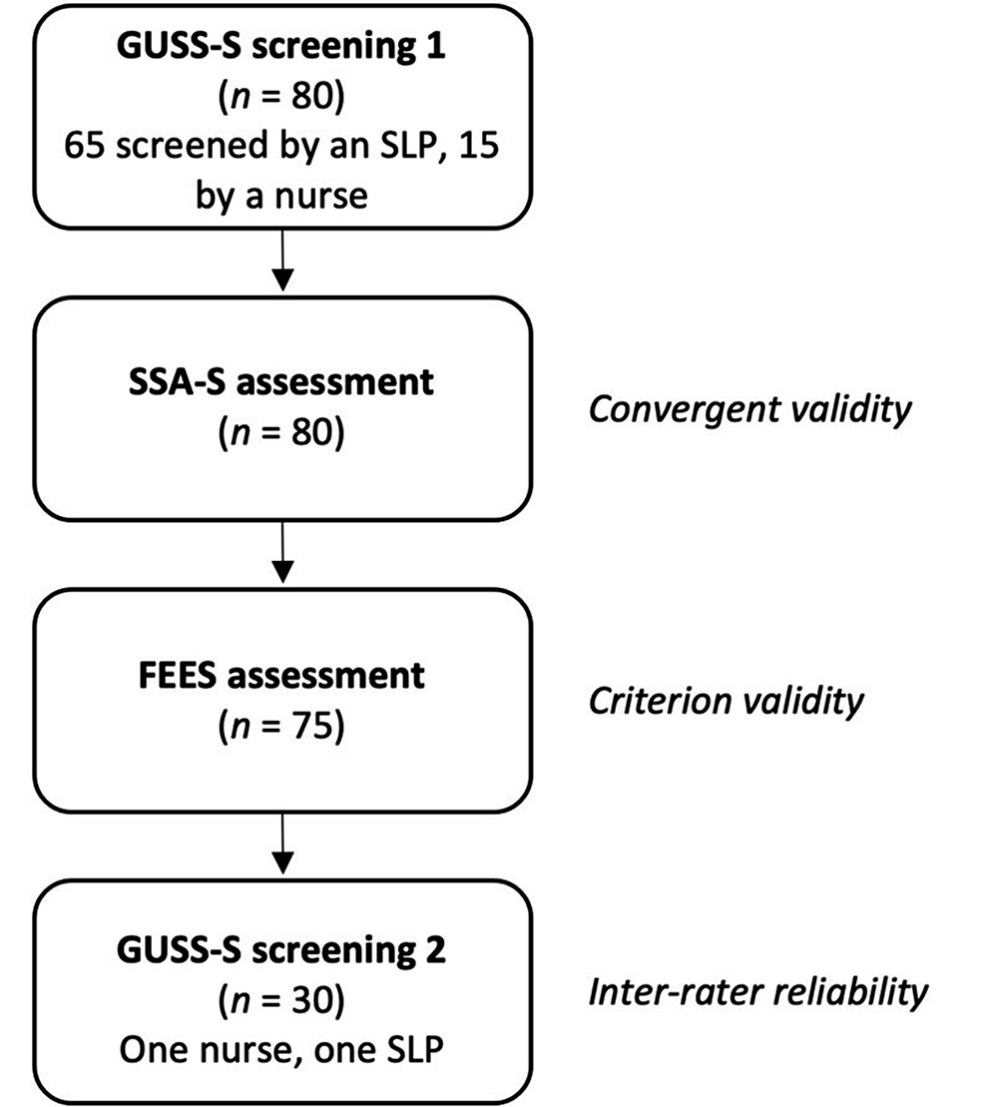
Flow chart of validation procedure. *Abbreviations* GUSS-S = The Gugging Swallowing Screen (Swedish), SLP = Speech-Language Pathologist, SSA-S = Standardized Swallowing Assessment (Swedish), FEES = Flexible Endoscopic Evaluation of Swallowing

This was a prospective cross-sectional study where the GUSS was translated into Swedish and validated in a Swedish stroke care context. The study was approved by the Swedish Ethical Review Authority (Dnr: 2019–06546, 2021–05043, 2021-06701-02) and conducted following the STARD guidelines [19], see appendix A and the Helsinki Declaration [20]. The study consisted of two parts. In the first part, the GUSS was translated into Swedish to evaluate the content validity of the translated screening tool (see Fig. 1). In the second part (see Fig. 2), the criterion validity of the translated GUSS was explored by comparing the results of screening with FEES results. Further, to obtain convergent validity, the GUSS results were compared with (a) the Functional Oral Intake Scale (FOIS) [21] as per the comprehensive FEES assessment, (b) the Standardized Swallowing Assessment (SSA) [11–13], and (c) the National Institutes of Health Stroke Scale (NIHSS) [22, 23]. To evaluate the inter-rater reliability of the translated GUSS, it was used independently by different healthcare professionals in examinations conducted within a period of 2 h. A further aim was to compare the SSA results with the FEES results, since the SSA screening tool has, to the best of the authors’ knowledge, not been validated against the gold standard assessment in an acute stroke population.

### Translation Methodology

The GUSS was translated using a forward-backward multistep approach as recommended by the World Health Organization [24]. The translation process consisted of seven phases. Continuous documentation of all the phases was made during the translation process.

#### Phase 1: Forward Translation

In the first phase, the forward translation from English into Swedish, two translators actively working in the field of dysphagia who were proficient and fluent in written English, independently translated the GUSS into Swedish.

#### Phase 2: Expert Panel

Phase two consisted of a review of the two translated versions by an expert panel of nine healthcare professionals working in the field of dysphagia (four SLPs, two nurses, two dietitians, and one otolaryngologist) to identify any discrepancies between, and specific difficulties with, the original scale and the two parallel translations.

#### Phase 3: Review

In the third step, the two forward translators and another reviewer who had not participated in the expert panel, reviewed the expert panel’s opinions of the two parallel translations in relation to the original version of GUSS. The goal of the third step was to decide on a final version of GUSS-S. The review group and the last author made the final decision regarding the consensus version of the GUSS-S.

#### Phase 4: Back-translation

A bi-lingual translator with experience of working with dysphagia and blinded to the original version of the GUSS back-translated the final version of GUSS-S.

#### Phase 5: Review

The back-translation was then compared with the original version of the GUSS by the same review panel as in Phase 3. The goal of the comparison was to identify any discrepancies between the original version and the back translation.

#### Phase 6: Pretesting

The GUSS-S was pretested in two ways. First, an expert panel of twelve SLPs with experience of working with dysphagia were recruited via convenience sampling. The panel members each rated the translation regarding relevance for screening dysphagia in a Swedish context to enable calculation of the content validity of the GUSS-S [25]. The ratings were collected via a digital survey platform. The SLPs rated seven items of the translated screening tool on a scale from 1 (not relevant) to 4 (very relevant). The ratings were used to measure inter-rater agreement through the calculation of a content validity index (CVI) [25]. Item-CVI (I-CVI) and CVI for the screening (Scale-CVI, S-DVI/Ave) were calculated according to convention. For each item, the number of experts who rated an item as a 3 or 4 was divided by the number of experts in total (*n* = 12). This gave the proportion in agreement concerning the relevance of each item. Items with an I-CVI of 0.78 or higher are considered to have excellent content validity when the panel consists of 3 or more experts [26]. All items reached excellent content validity since all I-CVI surpassed the limit of 0.78, with values varying between 0.83 and 1.00. Four items were rated with an I-CVI of 1.00, which means that all experts rated the items as relevant for screening dysphagia. S-CVI/Ave was computed by calculating the mean value of all I-CVI. S-CVI/Ave for the screening as a whole was 0.95, which is deemed excellent [25]. These results indicate that the GUSS-S has good-to-excellent CVI per item and an excellent average CVI. There was, therefore, no indication that further revision was needed before using the screening in a Swedish clinical care context.

The GUSS-S was then tested independently by a nurse and a SLP in the screening of five patients admitted to a stroke unit.

#### Phase 7: Validation

The final phase included validating the GUSS-S in a Swedish stroke care context.

The validation process is described in detail in Fig. 2.

### Validation Methodology

#### Participants

**Fig. 3:**
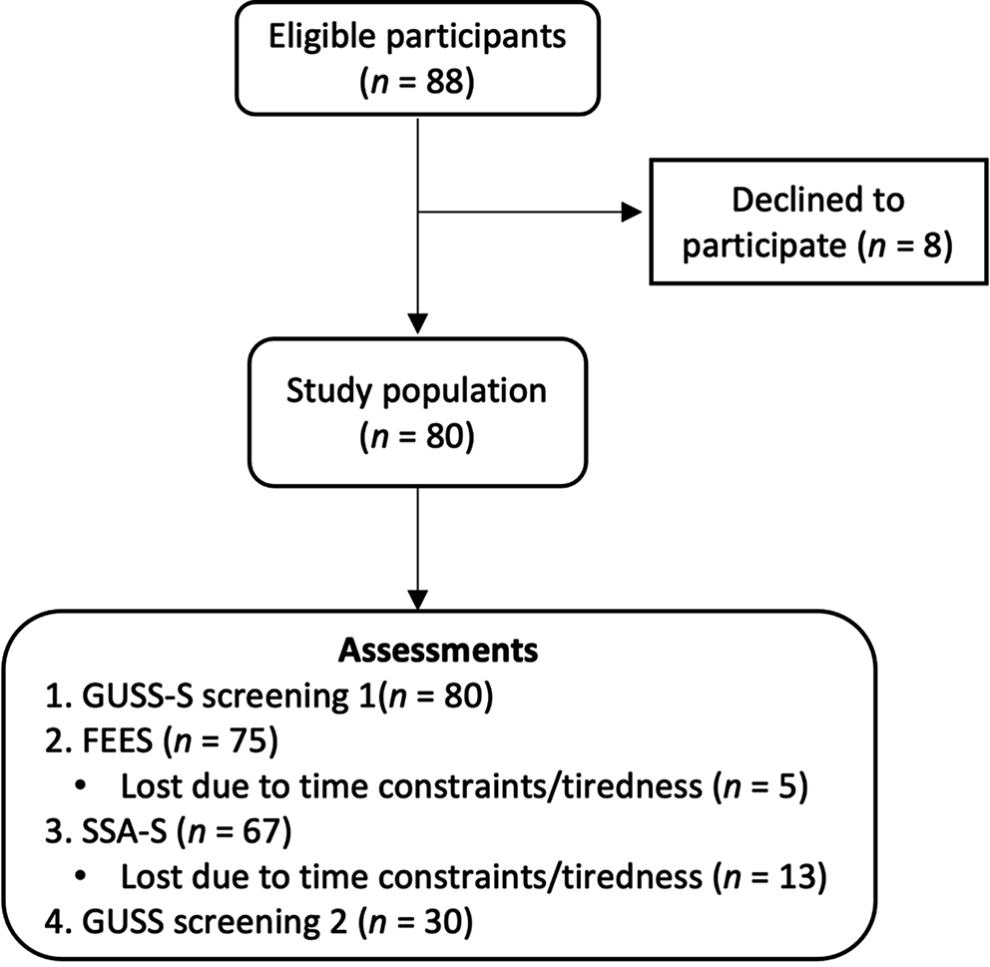
Flow-chart of subject inclusion and data collection, all performed within two hours. *Abbreviations* GUSS-S = The Gugging Swallowing Screen (Swedish), FEES = Flexible Endoscopic Evaluation of Swallowing, SSA-S = Standardized Swallowing Assessment (Swedish)

The validation was carried out on the stroke care unit at Umeå University Hospital. During the study period, a total of 88 patients were eligible to participate. Inclusion criteria were patients diagnosed with a stroke (i.e., ischemic stroke or intracerebral hemorrhagic stroke) or Transient Ischemic Attack (TIA), with no history of dysphagia, and that the assessments could be performed within 96 h of stroke/TIA onset. Eight patients were excluded due to (a) not being diagnosed with a TIA or stroke (*n* = 4), (b) not giving consent to be examined with FEES (*n* = 2), and (c) having an unstable medical condition that posed a contraindication for FEES (*n* = 2). A total of 80 patients were included in the validation process. Before inclusion, the patients signed an informed consent form or agreed to participate verbally if unable to sign by hand. See Fig. 3 for a flow-chart of the subject inclusion and data collection.

### GUSS Assessment

The patients were screened using GUSS-S within 96 h of admission to the stroke care unit. The GUSS-S screening was performed in accordance with the original publication by Trapl et al. [15]. The GUSS is divided into two parts: a preliminary assessment and a direct swallowing test consisting of three subtests that are performed sequentially. Each subtest gives a maximum of five points; a score of five points must be achieved to continue to the next subtest. A patient can attain a maximum of 20 points, which denotes normal swallowing ability. Nineteen to 15 points indicates mild dysphagia with a low or minimal risk of aspiration, 14 to10 points indicates moderate dysphagia with risk of aspiration, and 9 to 0 points indicates severe dysphagia with a high risk of aspiration.

Patients were assessed while sitting in an upright position. The preliminary assessment evaluated patients regarding vigilance, voluntary cough, throat clearing, and saliva swallowing. Those who scored five points in this assessment moved on to the second part. Those who scored below five were noted as having a risk of aspiration and testing was terminated. The second part started with a semi-solid swallowing trial where the patient was given water thickened to a consistency of 3 on the IDDSI framework [27]. The patient was first given half a teaspoon followed by an additional 3–5 teaspoons while the investigator observed the patient closely and scored according to the presence or absence of the following signs of aspiration: delayed (> 2 s) or impaired swallowing, voice change, drooling, and cough. If no aspiration signs were detected, the investigator would score accordingly and move on to liquid swallowing. The patient was given increasing amounts of water (IDDSI 0 [27]), starting with 3 ml and, after successful swallowing, continuing to 5, 10, 20 and 50 ml. In the final solid swallowing trial, the patient was instructed to ingest a small piece of dry bread (IDDSI 7 [27]); the time limit for delayed swallowing was ten seconds.

In this study, GUSS-S screening was first performed in 65 of 80 patients by an SLP and in 15 patients by a nurse. In 30 participants of the 65 screened by an SLP, a nurse performed a second screening with GUSS-S within 2 h of the first screening to assess inter-rater reliability. All performers of the GUSS-S were aware of clinical information about the patient from the medical records, but were blinded to all other assessments carried out in the validation procedure.

### FEES Assessment

FEES was used as the reference standard for validating the screening tool since it is considered (a) the gold standard for detecting dysphagia and (b) can be performed at bedside [28–30]. The FEES assessment was performed at bedside within 2 h of the GUSS-S screening on the stroke unit according to a standardized protocol by Langmore [31]. Two experienced SLPs performed the FEES. The examiners were blinded to the patient’s GUSS-S results and their medical records. Before the FEES assessment, one SLP performed the SSA [11–13] for convergent validity testing. The other SLP, blinded to the SSA result, performed the FEES and assessed the patient’s swallowing function. The FEES examination was carried out using a rhino-laryngo video endoscope (ENF-VH, Olympus), a light source (Visera Elite CLV-S190, Olympus), a video processor (OTV-S190, Olympus) and a video monitor (Olympus OEV143). Three bolus consistencies were tested during the FEES, the same as used in the GUSS-S screening: levels 0, 3 and 7 according to the IDDSI framework [27]. The bolus was dyed with green food coloring to enable visibility during the examination. Three teaspoons and three tablespoons of semi-solids (IDDSI 3) and liquid (IDDSI 0) were given in a consecutive order. The patient was then instructed to drink 2–3 mouthfuls of liquid (IDDSI 0) and to masticate and swallow a piece of cracker (IDDSI 7). The protocol was modified or shortened if the SLP determined that the patient’s safety was at risk due to significant aspiration or other severe dysphagia symptoms.

Based on the FEES findings, aspiration risk was classified according to the Penetration-Aspiration Scale (PAS) [32] with a cut-off point of ≥ 5, as per the original publication [15]. The presence of dysphagia was classified according to the Fiberoptic Endoscopic Dysphagia Severity Scale (FEDSS) scoring system [33], as per the revisited publication by Warnecke et al. [17]. The cut-off point of FEDSS > 1 was defined as presence of dysphagia. Based on the comprehensive FEES assessment, the examiner also rated the patient’s ability regarding intake of food and drink according to FOIS.

All assessments (GUSS, SSA, FEES, including FOIS) were performed within a period of 2 h and within 96 h of admission to the stroke unit (see Fig. 2).

### SSA-S Assessment

Similarly to the GUSS-S, the SSA-S consists of a preliminary investigation and a secondary direct swallowing test. In the first part, the patient’s vigilance and ability to sit upright and maintain adequate control of the head is assessed. The preliminary investigation also evaluates voluntary cough, control of saliva, mobility of the tongue, breathing, and quality of voice with subsequent termination of screening and SLP referral if one aspect or more is noted to be impaired. In the direct swallowing test, the patient is given teaspoons of water while the investigator observes the following signs of aspiration: absence of swallowing movements, drooling, coughing or shortness of breath, and “gurgly” voice after swallowing. If no signs of aspiration are noted, three teaspoons of water are administered, and the screening ends with the patient drinking half a glass of water.

### Statistical Analysis

All statistical analyses were performed using SPSS 28.0 Statistical Package for the Social Sciences [34]. Criterion validity was calculated for sensitivity and specificity in (a) percent and (b) based on the receiver operating characteristic curves (ROC), with the area under the curve being calculated. The analyses were based on the cut-off set of GUSS ≤ 14 points and PAS ≥ 5 for aspiration. For the presence of dysphagia, the analyses were based on the cut-off set of GUSS ≤ 19 points and FEDSS > 1. Convergence validity was calculated with Spearman rank correlation by analyzing the correlation between GUSS scores with PAS, FOIS, SSA, and NIHSS. Inter-rater reliability was analyzed using Cohen’s weighted kappa with quadratic weights. Sensitivity and specificity calculations were also performed in percent for dysphagia (yes/no), identified with SSA, compared with FEES, and assessed with the PAS cut-off ≥ 5.

## Results

### Participants

Of the 80 participants, 32 were women and the median age was 77 years (range 29–93). The participants had a median NIHSS score of 3 (range 0–21). The GUSS-S screening and the FEES assessment were performed at a mean of 1.7 ± 0.9 days after stroke/TIA onset. A total of 75 participants underwent FEES assessment. No adverse events occurred during any of the assessments in the study. For patient characteristics, see Table 1.

**Table 1:** Epidemiological and subject characteristics (*n =* 80)

Age median (range)	77 (29–93)
Sex	
Women/men	32/48
Ischaemic stroke/intracerebral hemorrhage (*n*)	65/6
Supratentorial location	43
Infratentorial location	25
Combined and multiple	3
TIA (*n)*	9
NIHSS *median / mean*	3/5.49
Medical history (*n*)	
Diabetes mellitus	17
Hypertension	48
Arrythmia	20
Hyperlipidemia	7
Smoking	4
Obesity	11
Obstructive sleep apnea	8

*Abbreviations* TIA = Transient Ischemic Attack, NIHSS = National Institutes of Health Stroke Scale

### GUSS-S and FEES Assessment

According to the GUSS-S screening performed by a SLP, 30 of 65 patients (46.2%) were identified with dysphagia (GUSS-S ≤ 19); of these, 27 (41.5%) were identified as being at risk of aspiration (GUSS-S ≤ 14). Among those with a risk of aspiration, severe dysphagia (GUSS-S ≤ 9) was identified in 13 (20%) participants. According to the FEES assessment, 31 of 75 (41.3%) participants were classified as having dysphagia (FEDSS score > 1). A total of 16 of 75 (21.3%) participants were classified as aspirating (PAS score ≥ 5).

### Criterion Validity

**Fig. 4:**
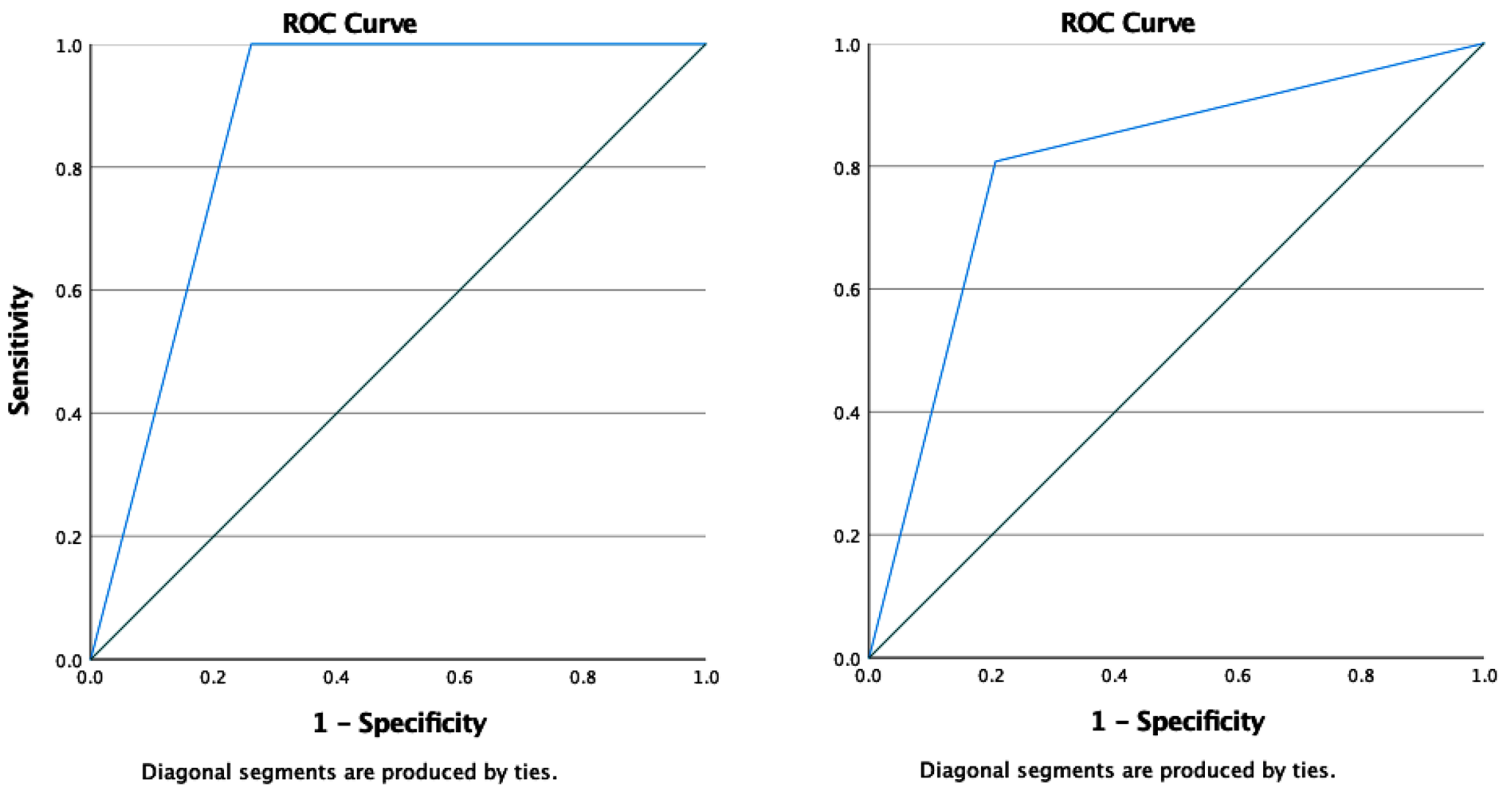
Receiver-Operator Characteristics Curves (ROC) for the Swedish version of the Gugging Swallowing Screen (GUSS-S) with a cut-off set to ≤ 14 compared to Flexible Endoscopic Evaluation of Swallowing (FEES) analyzed with the Penetration-Aspiration Scale (PAS) and a cut-off set to ≥ 5 (left), and for the GUSS-S with a cut-off set to ≤ 19 compared to FEES analyzed with Fiberoptic Endoscopic Dysphagia Severity Scale (FEDSS) and a cut-off set to > 1 (right)

The criterion validity of the GUSS-S for screening for aspiration (GUSS-S ≤ 14) and dysphagia (GUSS-S ≤ 19) are shown in Table 2. With the GUSS cut-off value of 14 points, as per the original validation, the GUSS-S identified aspiration with a sensitivity of 100% and a specificity of 73.9% (area under the curve was 0.87 (CI = 95%, 0.78–0.95); see Fig. 4).

**Table 2:** Sensitivity, specificity and predicative values in the Swedish version of the GUSS compared to gold standard FEES assessment evaluated with PAS and FEDSS (*n* = 60)

	*FEES results*		
	Aspiration Risk (PAS 5–8)	No Aspiration Risk (PAS 1–4)	
*GUSS results*			
Aspiration Risk (0–14)	14	12	PPV = 100% (N/A)
No Aspiration Risk (15–20)	0	34	NPV = 53.8% (0.35–0.72)
Sensitivity	100% (N/A)	Specificity	73.9% (0.60–0.85)
AUC total	0.87 (0.78–0.95)	Prevalence	76.7% (0.45–0.70)
	*FEES results*		
	Dysphagia (FEDSS 2–6)	Normal Swallowing (FEDSS > 1)	
*GUSS results*			
Dysphagia (0–19)	21	7	PPV = 75% (0.57–0.88)
Normal Swallowing (20)	5	27	NPV = 84.4%
Sensitivity	80.8% (0.63–0.92)	Specificity	79.4% (0.64–0.90)
AUC total	0.80 (0.68–0.95)	Prevalence	56.7% (0.46–0.69)

*Abbreviations* FEES = Flexible Endoscopic Evaluation of Swallowing, PAS = Penetration-Aspiration Scale, GUSS = The Gugging Swallowing Screen, FEDSS = Fiberoptic Endoscopic Dysphagia Severity Scale. AUC = Area Under the Curve. PPV = Positive Predicative Value. NPV = Negative Predicative Value. N/A = Not Applicable. Results are presented descriptively or as percentages (95% CI)

### Convergent Validity

The GUSS-S showed a very strong correlation with the FOIS as per the comprehensive FEES assessment (r_s_ = 0.720, *P* = < 0.001) and PAS (r_s_ = -0.718, *P* = < 0.001). A strong correlation was also obtained between the GUSS-S and the SSA-S (r_s_ = 0.545, *P* = < 0.001) and NHISS (r_s_ = -0.447, p = < 0.001).

When the SSA was compared with FEES, the screening tool identified aspiration with a sensitivity of 63.6% and a specificity of 75% (area under the curve was 0.69 (CI = 95%, 0.51–0.87), and dysphagia with a sensitivity of 56% and a specificity of 83.3% (area under the curve was 0.69 (CI = 95%, 0.56–0.83).

### Inter-Rater Reliability

Thirty patients were assessed twice using the GUSS-S within two hours, once by a SLP and once by a nurse. The inter-rater reliability between the two healthcare professionals showed substantial agreement (_Kw_ = 0.672, *P* = < 0.001 as computed using Cohen’s weighted Kappa with quadratic weights).

## Discussion

This study assessed the cross-cultural validity and inter-rater reliability of the Swedish version of the Gugging Swallowing Screen, GUSS-S for use in acute stroke care. Content validity of GUSS-S was calculated based on ratings by an expert panel. Criterion validity was evaluated by comparing GUSS-S results with FEES results, and convergent validity by comparing GUSS-S results with SSA-S, FOIS and NIHSS results. Inter-rater reliability was assessed between a SLP and a nurse when using GUSS-S in a Swedish stroke care context. Results indicate that the GUSS-S has excellent validity, as well as substantial inter-rater reliability when different healthcare professionals use this for screening. To the best of the authors’ knowledge, this is the first time the screening tool’s rater reliability when used by different healthcare professionals has been evaluated. The results will be further discussed below in relation to previous research and clinical applications.

The GUSS-S showed excellent criterion validity for predicting aspiration, with 100% sensitivity and 74% specificity, when compared to assessment with FEES using the PAS. It showed an 81% sensitivity and 79% specificity for predicting dysphagia. Furthermore, the GUSS-S yielded strong convergence validity with FOIS as per the comprehensive FEES assessment and the PAS, and strong convergence validity with the SSA and the NIHSS. The GUSS-S thus surpassed acceptable values for both sensitivity and specificity (70% and 60%, respectively) for detecting both dysphagia and aspiration, as suggested by Bours et al. [9].

The high sensitivity in the current study is in agreement with previous studies investigating the validity of GUSS when used in a stroke context [18, 35, 36] and other dysphagia screening tools used in stroke patients [37–42]. However, the specificity is higher in the current study compared to the original [15] and the revisited [17] publications. In the original publication of the GUSS, Trapl et al. [15] found a sensitivity of 100% and a specificity of 50 to 69% for predicting aspiration. In the revisited version of GUSS, Warnecke et al. [17] observed similar results (97% sensitivity and 56% specificity) although this study used a different FEES outcome scale than the original publication. For comparability between studies, the current study used the PAS, the same outcome scale for predicting aspiration as used in the original publication, and the FEDSS, the same outcome scale as used in the revisited publication, for predicting dysphagia. High sensitivity is essential in the acute phase of stroke since a dysphagia screening tool aims to identify patients with a risk of aspiration and to minimize respiratory infections, i.e., pneumonia [43]. The GUSS-S therefore succeeds extremely well in the identification of patients at risk of aspiration.

The higher specificity in our study compared to the original [15] and the revisited [17] publications might be explained by methodological differences between the studies. For example, the time from stroke onset to (a) assessment varied from 24 h in the original study, 72 h in the revisited, to 96 h in the current study, (b) completed assessments varied from within 2 h in the current study and the original, to 72 h in the revisited publication, and (c) the tested population was different. The current study included patients diagnosed with either TIA or stroke, while the original publication only included patients diagnosed with stroke and the revisited publication included patients with more severe stroke (NIHSS > 3). In the revisited study by Warnecke et al. [17], the specificity decreased with a higher NIHSS score. The authors suggested that a possible explanation for these results is in part because patients with an NIHSS score higher than 15 might often not manage the first part of screening using GUSS. The authors further suggested that a direct instrumental assessment, such as FEES, may be more appropriate in these patients (NIHSS ≥ 15) than dysphagia screening assessment during the acute phase of stroke onset. The explanation given by Warnecke et al. [17] might thus reflect why a higher specificity was reached in the current study since patients with milder strokes were included. Despite this, Warnecke et al. [33] also highlighted that the validity of the GUSS should be investigated in patients with minor strokes. The current study’s evaluation of the screening of patients with TIA or stroke reflects the clinical routine and environment. According to national guidelines, a dysphagia screening test should be performed on all patients with suspected TIA or stroke who are admitted to hospital [4]. Consequently, a dysphagia screening tool used for such cases should be tested on the target population. Other studies where GUSS was tested in a varied dysphagia population have also shown high specificity. AbdelHamid and Abo-Hasseba [16] tested the Egyptian version of GUSS in 42 patients with complaints of dysphagia in an outpatient clinic and reached a sensitivity and specificity of 93% and 83%, respectively.

The inter-rater reliability in the current study reached substantial agreement between testing by a SLP and a nurse using GUSS-S, suggesting it is a reliable tool for nursing staff to use in a stroke and TIA population. This result is in congruence with previous studies of GUSS where SLPs or physicians have used the screening [15, 16, 35], and indicates that the screening tool is useful for screening by healthcare professionals other than SLPs. However, based on our results, structured training in screening for dysphagia in the early stages of stroke could be beneficial, not only in maintaining sensitivity and further improving inter-rater reliability between SLPs and nurses, but also for improving the health outcomes of the patient, as found in previous studies [44].

An interesting secondary finding in the present study was that the water-swallowing test SSA-S did not surpass sensitivity values in dysphagia screening compared to PAS (aspiration) or FEDSS (dysphagia). This result suggests that a water-swallowing test such as SSA-S could pose a risk for both over and underdiagnosing, and a step-wise screening using a wider range of consistencies might be preferred for use in an acute stroke population.

The results from the current study thus indicate that the GUSS-S is a valid and reliable screening tool for detecting both aspiration and dysphagia among acute stroke patients in a Swedish context. Results from this and other studies support the contention that the GUSS screening tool is appropriate for use by different healthcare professionals working on a stroke unit. These results are of great value since the screening can be implemented in clinical and research settings for stroke care.

### Limitations

As with all research, this study has its limitations. Although assessment within 96 h is considered during the acute phase, the screening should be applied to patients within 24 h of stroke onset or before oral intake of food or drink according to guidelines [3]. The incidence of dysphagia will also likely be affected by delayed screening. The sample size was calculated based on the number of items rated for content validity of the GUSS-S, but not on a dedicated power calculation. The current study included patients with a range of stroke severity, from mild to severe, but with the majority being mild. Future studies should include a larger sample with various stroke severity to further investigate the tool’s validity. Future studies should also address the intra-rater reliability of the GUSS-S, which has not been tested in the present study as the study population and protocol did not make blinding and repeated tests feasible.

## Conclusion

GUSS-S was produced after translation according to international guidelines and validated prospectively in a Swedish context among patients with acute stroke by various healthcare professionals commonly working with dysphagia, such as SLPs, nurses, dietitians, and physicians. The results demonstrated substantial agreement between a SLP and a nurse in assessments with the GUSS-S, indicating that it can be used by different healthcare professionals. The validity of the GUSS-S was excellent and thus the screening tool is valid and reliable to identify aspiration and dysphagia in the acute phase of stroke in a Swedish context.

## Electronic Supplementary Material

Below is the link to the electronic supplementary material.

Supplementary Material 1

## Data Availability

Data supporting the results and analyses are available on request from the final author (PH).

## References

[1] Martino R, Foley N, Bhogal S, Diamant N, Speechley M, Teasell R. Dysphagia after Stroke Stroke. 2005;36(12):2756–63. 16269630 10.1161/01.STR.0000190056.76543.eb

[2] Cohen DL, Roffe C, Beavan J, Blackett B, Fairfield CA, Hamdy S, et al. Post-stroke dysphagia: a review and design considerations for future trials. Int J Stroke. 2016;11(4):399–411. 27006423 10.1177/1747493016639057

[3] Dziewas R, Michou E, Trapl-Grundschober M, Lal A, Arsava EM, Bath PM, et al. European Stroke Organisation and European Society for Swallowing Disorders guideline for the diagnosis and treatment of post-stroke dysphagia. Eur Stroke J. 2021;6(3):LXXXIX–CXV. 34746431 10.1177/23969873211039721 PMC8564153

[4] The Swedish National Board of Health and Welfare. National Guidelines for Stroke Care - Support for Governance and Management. Internet: Socialstyrelsen; 2020 [cited 20231219]. https://www.socialstyrelsen.se/globalassets/sharepoint-dokument/artikelkatalog/nationella-riktlinjer/2020-1-6545.pdf.

[5] Foley J, Nund RL, Ward EC, Burns CL, Wishart LR, Graham N, et al. Clinician and consumer perceptions of head and neck cancer services in rural areas: implications for speech pathology service delivery. Aust J Rural Health. 2022;30(2):175–87. 35064946 10.1111/ajr.12829

[6] Palli C, Fandler S, Doppelhofer K, Niederkorn K, Enzinger C, Vetta C, et al. Early Dysphagia screening by trained nurses reduces Pneumonia Rate in Stroke patients. Stroke. 2017;48(9):2583–5. 28716980 10.1161/STROKEAHA.117.018157

[7] Baijens LW, Clavé P, Cras P, Ekberg O, Forster A, Kolb GF, et al. European Society for Swallowing Disorders - European Union Geriatric Medicine Society white paper: oropharyngeal dysphagia as a geriatric syndrome. Clin Interv Aging. 2016;11:1403–28. 27785002 10.2147/CIA.S107750 PMC5063605

[8] Rao N, Brady SL, Chaudhuri G, Donzelli JJ, Wesling MW. Gold-Standard? Analysis of the videofluoroscopic and fiberoptic endoscopic swallow examinations. J Appl Res. 2003;3(1):89.

[9] Bours GJ, Speyer R, Lemmens J, Limburg M, de Wit R. Bedside screening tests vs. videofluoroscopy or fibreoptic endoscopic evaluation of swallowing to detect dysphagia in patients with neurological disorders: systematic review. J Adv Nurs. 2009;65(3):477–93. 19222645 10.1111/j.1365-2648.2008.04915.x

[10] Boaden E, Burnell J, Hives L, Dey P, Clegg A, Lyons MW, et al. Screening for aspiration risk associated with dysphagia in acute stroke. Cochrane Database Syst Rev. 2021;10(10):Cd012679. 34661279 10.1002/14651858.CD012679.pub2 PMC8521523

[11] Alvarez C, Lai D. Validering av The Standardized Swallowing Assessment – Svenska (SSA-S) i screening av dysfagi vid akut stroke. 2014.

[12] Perry L. Screening swallowing function of patients with acute stroke. Part two: detailed evaluation of the tool used by nurses. J Clin Nurs. 2001;10(4):474–81. 11822495 10.1046/j.1365-2702.2001.00502.x

[13] Perry L. Screening swallowing function of patients with acute stroke. Part one: identification, implementation and initial evaluation of a screening tool for use by nurses. J Clin Nurs. 2001;10(4):463–73. 11822494 10.1046/j.1365-2702.2001.00501.x

[14] Doggett DL, Tappe KA, Mitchell MD, Chapell R, Coates V, Turkelson CM. Prevention of Pneumonia in Elderly Stroke patients by systematic diagnosis and treatment of Dysphagia: an evidence-based Comprehensive Analysis of the literature. Dysphagia. 2001;16(4):279–95. 11720404 10.1007/s00455-001-0087-3

[15] Trapl M, Enderle P, Nowotny M, Teuschl Y, Matz K, Dachenhausen A, et al. Dysphagia bedside screening for acute-stroke patients: the gugging swallowing screen. Stroke. 2007;38(11):2948–52. 17885261 10.1161/STROKEAHA.107.483933

[16] AbdelHamid A, Abo-Hasseba A. Application of the GUSS test on adult Egyptian dysphagic patients. Egypt J Otolaryngol. 2017;33(1):103–10.

[17] Warnecke T, Im S, Kaiser C, Hamacher C, Oelenberg S, Dziewas R. Aspiration and dysphagia screening in acute stroke - the gugging swallowing screen revisited. Eur J Neurol. 2017;24(4):594–601. 28322006 10.1111/ene.13251

[18] Umay E, Eyigor S, Karahan AY, Gezer IA, Kurkcu A, Keskin D, et al. The GUSS test as a good indicator to evaluate dysphagia in healthy older people: a multicenter reliability and validity study. Eur Geriatr Med. 2019;10(6):879–87. 34652777 10.1007/s41999-019-00249-2

[19] Bossuyt PM, Reitsma JB, Bruns DE, Gatsonis CA, Glasziou PP, Irwig L, et al. STARD 2015: an updated list of essential items for reporting diagnostic accuracy studies. BMJ. 2015;351:h5527. 26511519 10.1136/bmj.h5527 PMC4623764

[20] World Medical A. World Medical Association Declaration of Helsinki: ethical principles for medical research involving human subjects. JAMA. 2013;310(20):2191–4. 24141714 10.1001/jama.2013.281053

[21] Crary MA, Mann GD, Groher ME. Initial psychometric assessment of a functional oral intake scale for dysphagia in stroke patients. Arch Phys Med Rehabil. 2005;86(8):1516–20. 16084801 10.1016/j.apmr.2004.11.049

[22] Brott T, Adams HP, Olinger CP, Marler JR, Barsan WG, Biller J, et al. Measurements of acute cerebral infarction: a clinical examination scale. Stroke. 1989;20(7):864–70. 2749846 10.1161/01.str.20.7.864

[23] Goldstein LB, Samsa GP. Reliability of the National Institutes of Health Stroke Scale. Stroke. 1997;28(2):307–10. 9040680 10.1161/01.str.28.2.307

[24] Organisation WH. Process of translation and adaptation of instruments. Internet2019 [cited 2023 0306]. http://www.who.int/substance_abuse/research_tools/translation/en/.

[25] Polit DF, Beck CT, Owen SV. Is the CVI an acceptable indicator of content validity? Appraisal and recommendations. Res Nurs Health. 2007;30(4):459–67. 17654487 10.1002/nur.20199

[26] Shi J, Mo X, Sun Z. [Content validity index in scale development]. Zhong Nan Da Xue Xue Bao Yi Xue Ban. 2012;37(2):152–5. 22561427 10.3969/j.issn.1672-7347.2012.02.007

[27] Cichero JA, Lam P, Steele CM, Hanson B, Chen J, Dantas RO, et al. Development of International Terminology and definitions for texture-modified foods and Thickened fluids used in Dysphagia Management: the IDDSI Framework. Dysphagia. 2017;32(2):293–314. 27913916 10.1007/s00455-016-9758-y PMC5380696

[28] Langmore SE. Evaluation of oropharyngeal dysphagia: which diagnostic tool is superior? Curr Opin Otolaryngol Head Neck Surg. 2003;11(6):485–9. 14631184 10.1097/00020840-200312000-00014

[29] Langmore SE. History of fiberoptic endoscopic evaluation of swallowing for evaluation and management of pharyngeal dysphagia: changes over the years. Dysphagia. 2017;32(1):27–38. 28101663 10.1007/s00455-016-9775-x

[30] Labeit B, Ahring S, Boehmer M, Sporns P, Sauer S, Claus I, et al. Comparison of simultaneous swallowing Endoscopy and Videofluoroscopy in Neurogenic Dysphagia. J Am Med Dir Assoc. 2022;23(8):1360–6. 34678269 10.1016/j.jamda.2021.09.026

[31] Langmore SE, Schatz K, Olsen N. Fiberoptic endoscopic examination of swallowing safety: a new procedure. Dysphagia. 1988;2(4):216–9. 3251697 10.1007/BF02414429

[32] Rosenbek JC, Robbins JA, Roecker EB, Coyle JL, Wood JL. A penetration-aspiration scale. Dysphagia. 1996;11(2):93–8. 8721066 10.1007/BF00417897

[33] Dziewas R, Warnecke T, Olenberg S, Teismann I, Zimmermann J, Kramer C, et al. Towards a basic endoscopic assessment of swallowing in acute stroke - development and evaluation of a simple dysphagia score. Cerebrovasc Dis. 2008;26(1):41–7. 18511871 10.1159/000135652

[34] IBM SPSS Statistics for Macintosh. Version 28.0. Armonk, NY: IBM Corp; 2021.

[35] Woo LK, Beom KS, Hwa LJ, Ah KM, Hee KB, Cheol LG. Clinical validity of gugging swallowing screen for Acute Stroke patients. Ann Rehabil Med. 2009;33(4):458–62.

[36] Szabó PT, Műhelyi V, Halász T, Béres-Molnár KA, Folyovich A, Balogh Z. Hungarian adaptation of an international swallowing screening method. Orv Hetil. 2022;163(36):1431–9. 36057872 10.1556/650.2022.32566

[37] Edmiaston J, Connor LT, Loehr L, Nassief A. Validation of a dysphagia screening tool in acute stroke patients. Am J Crit Care. 2010;19(4):357–64. 19875722 10.4037/ajcc2009961 PMC2896456

[38] Martino R, Silver F, Teasell R, Bayley M, Nicholson G, Streiner DL, et al. The Toronto Bedside swallowing screening test (TOR-BSST): development and validation of a dysphagia screening tool for patients with stroke. Stroke. 2009;40(2):555–61. 19074483 10.1161/STROKEAHA.107.510370

[39] Daniels SK, Pathak S, Rosenbek JC, Morgan RO, Anderson JA. Rapid Aspiration Screening for suspected stroke: part 1: development and validation. Arch Phys Med Rehabil. 2016;97(9):1440–8. 27117382 10.1016/j.apmr.2016.03.025

[40] Antonios N, Carnaby-Mann G, Crary M, Miller L, Hubbard H, Hood K, et al. Analysis of a physician tool for evaluating dysphagia on an inpatient stroke unit: the modified Mann Assessment of swallowing ability. J Stroke Cerebrovasc Dis. 2010;19(1):49–57. 20123227 10.1016/j.jstrokecerebrovasdis.2009.03.007

[41] Daniels SK, Anderson JA, Willson PC. Valid items for screening dysphagia risk in patients with stroke: a systematic review. Stroke. 2012;43(3):892–7. 22308250 10.1161/STROKEAHA.111.640946

[42] Schepp SK, Tirschwell DL, Miller RM, Longstreth WT. Jr. Swallowing screens after acute stroke: a systematic review. Stroke. 2012;43(3):869–71. 22156697 10.1161/STROKEAHA.111.638254 PMC3288702

[43] Walter U, Knoblich R, Steinhagen V, Donat M, Benecke R, Kloth A. Predictors of pneumonia in acute stroke patients admitted to a neurological intensive care unit. J Neurol. 2007;254(10):1323–9. 17361338 10.1007/s00415-007-0520-0

[44] Al-Khaled M, Matthis C, Binder A, Mudter J, Schattschneider J, Pulkowski U, et al. Dysphagia in patients with Acute ischemic stroke: early Dysphagia Screening May reduce stroke-related pneumonia and improve stroke outcomes. Cerebrovasc Dis. 2016;42(1–2):81–9. 27074007 10.1159/000445299

